# Multimodal learning for temporal relation extraction in clinical texts

**DOI:** 10.1093/jamia/ocae059

**Published:** 2024-03-26

**Authors:** Timotej Knez, Slavko Žitnik

**Affiliations:** Faculty of Computer and Information Science, University of Ljubljana, Ljubljana 1000, Slovenia; Faculty of Computer and Information Science, University of Ljubljana, Ljubljana 1000, Slovenia

**Keywords:** temporal relation extraction, knowledge graphs, natural language processing, transformer-architecture

## Abstract

**Objectives:**

This study focuses on refining temporal relation extraction within medical documents by introducing an innovative bimodal architecture. The overarching goal is to enhance our understanding of narrative processes in the medical domain, particularly through the analysis of extensive reports and notes concerning patient experiences.

**Materials and Methods:**

Our approach involves the development of a bimodal architecture that seamlessly integrates information from both text documents and knowledge graphs. This integration serves to infuse common knowledge about events into the temporal relation extraction process. Rigorous testing was conducted on diverse clinical datasets, emulating real-world scenarios where the extraction of temporal relationships is paramount.

**Results:**

The performance of our proposed bimodal architecture was thoroughly evaluated across multiple clinical datasets. Comparative analyses demonstrated its superiority over existing methods reliant solely on textual information for temporal relation extraction. Notably, the model showcased its effectiveness even in scenarios where not provided with additional information.

**Discussion:**

The amalgamation of textual data and knowledge graph information in our bimodal architecture signifies a notable advancement in the field of temporal relation extraction. This approach addresses the critical need for a more profound understanding of narrative processes in medical contexts.

**Conclusion:**

In conclusion, our study introduces a pioneering bimodal architecture that harnesses the synergy of text and knowledge graph data, exhibiting superior performance in temporal relation extraction from medical documents. This advancement holds significant promise for improving the comprehension of patients’ healthcare journeys and enhancing the overall effectiveness of extracting temporal relationships in complex medical narratives.

## Introduction

Electronic health records (EHRs) serve as crucial repositories of medical information, with both structured and unstructured data. While structured information supports automated analysis, vital details are often embedded in free-text notes.^1^

Efficient analysis of medical documents requires reconstructing events for chronological understanding.^2^ Recognizing temporal relations, particularly through temporal relation extraction, is pivotal for deciphering the sequence of events in patient care, aiding diagnosis, and informing treatment decisions.^2^

Current research on temporal relation extraction often incorporates statistical resources without fully exploring the potential of common knowledge integration. Existing approaches utilize statistics on common relations between events.^3^^,^^4^

This paper introduces a novel model for temporal relation extraction, leveraging a bimodal architecture that combines event embeddings from both the text and a knowledge graph. By incorporating common knowledge, the model enhances performance in determining the temporal order of events. The proposed model is trained and evaluated on clinical documents from the i2b2 2012 dataset,^5^ MACCROBAT2020 dataset,^6^ and THYME dataset.^7^

The code used in this paper is available on Github (https://github.com/TimotejK/Multi-modal-temporal-relation-extraction).

## Related work

Temporal relation extraction is a prevalent undertaking in the domain of event time recognition. Its primary objective is to establish the temporal association between the times at which 2 events transpired. For instance, a temporal relation would recognize that event A transpired “before” event B.

### Temporal relation extraction using traditional machine learning

The task of temporal relation extraction gained the attention of the research community with the introduction of the TimeBank corpus.^8^ The early approaches for solving this task were based on hand-defined rules.^9–11^ For example, Gaizauskas et al^9^ recognize temporal relations using rules based on simple features like verb tense and aspect. Such models were soon outperformed by models using machine learning techniques. The early machine-learning approaches for temporal relation extraction relied on hand-engineered features, classified by simple models. Such as maximum entropy (ME)^11–13^ and the support vector machine (SVM) models.^12^^,^^14^

### Deep neural networks

Recent systems predominantly rely on deep neural network architectures built upon either LSTM networks or pretrained language models.

LSTM-based models, exemplified by Tourille et al,^15^ Cheng and Miyao,^16^ and Leeuwenberg and Moens,^17^ employ LSTM layers to capture contextualized embeddings from text tokens. They extract relations through classification using a feed-forward network on combined embeddings from event mentions. Cheng and Miyao^16^ diverge by using LSTM networks over dependency paths instead of consecutive words. Leeuwenberg and Moens^17^ predict event times and durations directly from LSTM embeddings, allowing absolute event timelines but potentially lower prediction reliability.

In contrast, recent successful architectures rely on pretrained language models, as shown by Lin et al,^18^ Zhou et al,^19^ and Lin et al.^20^ Lin et al^18^ mark events with special tokens and use a BERT network, classifying the class token [CLS] embedding to determine temporal relations. Zhou et al^19^ enhance results with soft logic regularization, predicting relation probabilities and ensuring rule-based consistency. Lin et al^20^ improve medical domain extraction through entity-specific BERT pretraining.

Our research aims to enhance temporal relation prediction by building on Lin et al’s^20^ model, integrating common knowledge via an event graph input. Our architecture combines BERT for text embeddings with a graph neural network for event embeddings, culminating in a composite vector for the final temporal relation prediction.

### Improving relation extraction using additional knowledge

When tackling tasks like temporal relationship recognition, we go beyond text data and leverage common knowledge. For instance, we intuitively understand the temporal sequence of events, even if not explicitly mentioned, like dinner preceding sleep. To infuse this intuition into machine models for temporal relation extraction, we can utilize statistics compiled from a large corpus. Ning et al^3^ demonstrated this by creating a statistical resource based on New York Times articles spanning 20 years. They improved temporal relation extraction in the news domain by using this resource, encoding event pairs based on their semantics, resulting in a 3% performance gain.

To enhance this approach, we propose using event embeddings from a knowledge graph to incorporate additional information about events. We believe the most significant benefit will arise from considering temporal relations between related events within the same document, a facet not covered by Ning et al.^21^

### Multimodal learning

Recent research proposes bimodal models for combining text and knowledge graph data, as seen in domains similar to temporal relation extraction.^22–24^ Lin et al^22^ apply this approach to extract disease relations by encoding text with SciBERT and the knowledge graph with a heterogeneous graph attention network. They merge these encodings for final classification. Yasunaga et al^23^ introduce DRAGON, a general pretrained network that integrates text and knowledge graph data. The key difference lies in the fusion method: DRAGON combines modalities into embeddings, while Lin et al separately compute embeddings for each modality and combine them later. Our work employs a multimodal model similar to Lin et al’s,^22^ with adapted encoders for temporal relation extraction while preserving the idea of merging encodings for final classification.

## Methods

Our aim is to improve temporal relation extraction from text by introducing a novel model that leverages extra common knowledge via a knowledge graph. This model processes both text and a knowledge graph as inputs, merging them to discern the temporal relationships between identified events.

### Additional knowledge representation

In our experiments, we represent additional knowledge using knowledge graphs termed event graphs that contain events represented by nodes and their temporal relations represented as edges. In an event graph, we include some already known relations related to the events between which we are trying to predict the temporal relation, providing additional temporal context to the model.

### Constructing the knowledge graph

When building an event graph, we need to consolidate the references to the same event into a single node within our knowledge graph. We achieve this by using the SciSpacy library. The library connects the events with the UMLS terminology system.^25^ This linking process associates the events with specific medical concepts and provides corresponding identifiers. For instance, in the sentence, “In addition, the patient desired liposuction of varying areas as well,” where the phrase “liposuction of varying areas” is labeled as an event, SciSpacy links this event to the UMLS concept C0038640 (Suction Lipectomy). We then group all events related to the same medical concept into a single node within the graph.

**Figure 1.**
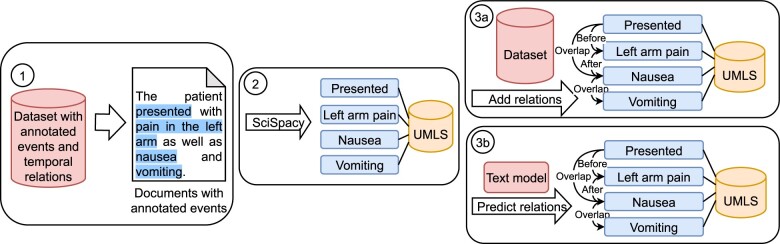
Preparation of a knowledge graph. (1) We get a document with annotated events from the dataset. (2) We use SciSpacy to disambiguate and link events to UMLS. (3) We add the temporal relations to the KG.

The SciSpacy library is only responsible for recognizing UMLS concepts from the event mentions which links the nodes in a knowledge graph to the UMLS terminology. For determining the edges of the graph corresponding to temporal relations, we designed 2 approaches described in sections “Scenario 1: using a full knowledge graph” and “Scenario 2: automatically inferred knowledge graph.” In the first approach, we use the relations recorded in the dataset that we augment using the pipeline described in section “Dataset preparation.” In the second approach, we predict the relations using the part of the model that relies solely on the text input. The process is shown in Figure 1. In this way we first build a single knowledge graph from all of the text in the dataset. After that we filter the graph for each relation prediction to keep only the part of the graph close to the target events (ie, no more than 3 edges away). This way we get a separate subgraph for each relation prediction. The subgraphs that are used in the classification always contain at least the nodes corresponding to the 2 target events. On average a subgraph used for a relation prediction contains 93 nodes and 463 edges.

### Model architecture

**Figure 2.**
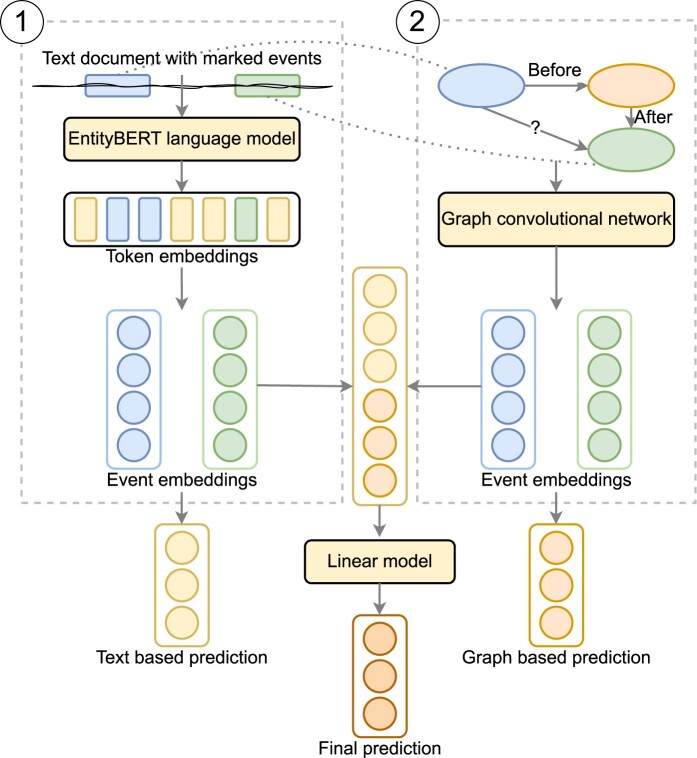
The architecture of the proposed system.

Our model architecture is inspired by Lin et al.^22^ The model’s structure is depicted in Figure 2 and comprises of 2 primary components, each responsible for processing a distinct input type. The first part (labeled 1 in Figure 2) encodes information from the text, while the second part (labeled 2 in Figure 2) extracts information from the knowledge graph. Each component generates a unique set of event embeddings. The model combines event embeddings to make a unified prediction. The separation of the 2 components allows us to compute predictions from a single modality when necessary.

### Text encoding

The text encoding module within the model (denoted as 1 in Figure 2) serves the purpose of extracting information from the text. The first step of extracting temporal relation is to mark the events by special tokens <e1>, </e1>, <e2>, and </e2>. Contextual embeddings of these text tokens are then computed using an EntityBERT model, as proposed by Lin et al.^20^

We aggregate the token embeddings associated with each event using mean. This results in 2 embeddings, 1 for each event. These embeddings are then concatenated. In cases where only the text model is used for temporal relation classification, the embeddings are passed through a feed-forward network for prediction. It is important to highlight that, despite the presence of special tokens marking the events in the text, we have observed through our experiments that utilizing the sentence embedding generated by the [CLS] token for classification achieved worse results.

### Graph encoding

The component identified as 2 in Figure 2 is responsible for encoding the knowledge graph information that accompanies the text input. To achieve this, we employ a graph neural network equipped with 3 convolutional layers. We formulated convolutional layers utilizing the methodology presented by Schlichtkrull et al,^26^ wherein distinct transformation matrices are learned for each relation type. In instances where graphs incorporate probability vectors denoting relation types, the convolutional operation calculates a weighted summation of all potential relation types according to the associated probability vector. To compute the initial node embeddings that the graph neural network propagates, we use glove embeddings of the event mention or the canonical name from the UMLS when available.

### Relation classification

After encoding both input parts, the model’s final phase classifies temporal relations by using the 2 pairs of event embeddings from the text and graph encoders. This step combines the last layers of both encoders and applies a single linear layer to produce the ultimate classification.

### Dataset preparation

We utilized various clinical datasets, as discussed in section “Datasets.” During training, we divided each dataset’s training segment into 2 training sets and 1 validation set. The predefined test portion of each dataset served for model evaluation.

**Figure 3.**
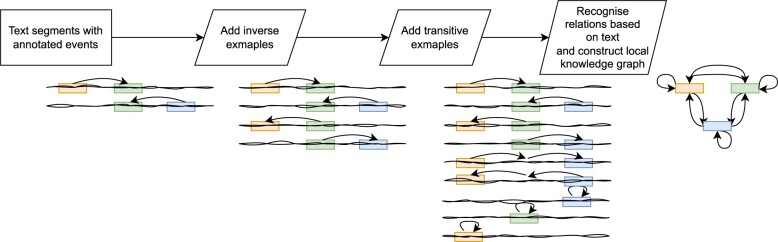
The pipeline for enriching the dataset and using it to construct an event graph. We start with some relations between the events annotated in the text. For each relation, we add an inverse relation if it is not already present. Then we add transitive relations.

In scenario 1 (see section “Scenario 1: using a full knowledge graph”), we employed dataset relations to construct an event graph, enhancing relation extraction. Additional relations were introduced algorithmically to improve graph completeness. We included inverse relations by reversing event orders for each original dataset relation. Subsequently, transitive relations were added based on simple rules. This process is illustrated in Figure 3.

### Datasets

In our experiments, we used 3 datasets containing medical documents annotated with events and their temporal relations. In Table 1 we present the parameters of the datasets used in the experiments. The numbers are split into 4 partitions of the dataset.

**Table 1.** Size comparison of the datasets used in the analysis of our model.

	Train	Train2	Val	Test	Sum
**THYME**					
Documents	110	40	49	101	300
Relations	10 542	3649	3586	9171	26 948
Events	12 640	4714	4511	11 265	33 130
**I2B2**					
Documents	117	32	40	120	309
Relations	8539	2841	2876	11 752	26 008
Events	8396	2829	2983	11 361	25 569
**MACCROBAT**					
Documents	90	31	24	55	200
Relations	4831	1610	1611	3455	11 507
Events	4612	1594	1522	11 361	19 089

A given threshold value means that we keep all relations with higher or equal softmax confidence.

#### *i*2*b*2 2012 *dataset*

The i2b2 2012 dataset^5^ for the 2012 i2b2 challenge includes 189 annotated training documents and 120 testing documents of medical discharge summaries. Temporal relations are categorized as before, after, or overlap, with 63.6%, 22.2%, and 14.2% distribution, respectively.

#### THYME dataset

The THYME dataset^7^^,^^27^ was created for SemEval 2016 with 300 documents related to cancer patients. It contains annotated events and temporal relations across 9 classes. We reclassified these into 3 categories (before, after, and overlap) for result comparability with other datasets. Testing was done on both modified and original annotations.

#### MACCROBAT dataset

The MACCROBAT dataset^6^^,^^28^ comprises 200 clinical case reports from PubMed Central, including 19 089 annotated events. It primarily serves a different purpose but also contains 11 507 temporal relations categorized as before, after, or overlap.

## Results

We conducted experiments to evaluate our model’s performance in constructing an event-centric knowledge graph and extracting temporal relations from textual data. Testing scenarios included using a full knowledge graph, an automatically inferred knowledge graph, and an ablation study.

### Scenario 1: using a full knowledge graph

We compared the model’s performance using only text input with a hybrid approach that combines text and a knowledge graph constructed from document relations, excluding those related to the target events. We used the relations that came from the same dataset and were close to the target events. We hypothesized that additional document relations would provide valuable temporal context for determining temporal relationships between target events. In this experiment, we simulated a scenario in which some relations were already available from a document, and the goal was to complement the existing knowledge graph with new relations.

The results, shown in Table 2, demonstrated that the hybrid approach outperformed text-only recognition across all datasets. The largest improvement was seen in the i2b2 and MACCROBAT datasets, with a smaller improvement in the THYME dataset, likely due to its complex relations.

**Table 2.** The results on the various datasets achieved by the model when provided with a graph containing relations from the observed document.

Dataset	Text*	Graph	**Bimodal** †	**Improvement** (†−*)
**Scenario 1: using a full knowledge graph**				
i2b2	73.11%	77.10%	**82.04%**	8.93%
THYME	72.41%	67.50%	**73.90%**	1.49%
THYME converted	72.69%	74.70%	**81.09%**	8.40%
MACCROBAT	58.01%	**82.69%**	81.54%	23.53%
**Scenario 2: automatically inferred knowledge graph**				
i2b2	73.11%	66.18%	**74.16%**	1.05%
THYME	**72.41%**	62.82%	69.79%	−2.62%
THYME converted	72.69%	54.80%	**73.12%**	0.43%
MACCROBAT	58.01%	**63.76%**	61.81%	3.81%
**Scenario 3: ablation study**				
i2b2	73.11%	71.88%	76.09%	2.98%
THYME	72.41%	65.56%	70.97%	−1.44%
THYME converted	72.69%	57.63%	72.82%	0.14%
MACCROBAT	58.01%	72.57%	72.83%	14.82%
**Models for comparison on i2b2 dataset**				
BERT base^29^	72.41%	/	/	/
BioBERT^29^	73.60%	/	/	/
Alpaca model	35.01%	/	/	/
**Models for comparison on THYME dataset**				
EntityBERT^20^	72.41%	/	/	/
Zhao et al^30^	64.3%	/	/	/
Wang et al^31^	66.1%	/	/	/
Tourille et al^15^	68.3%	/	/	/
Leeuwenberg et al^32^	62.8%	/	/	/

We test the model when using only the text part of the model, only the graph part of the model, and when using a combination of both inputs. The THYME converted dataset refers to the THYME dataset with all relations converted to 1 of the 3 i2b2 relations and over-sampled to remove class imbalance. We also included results from other papers for comparison. The most direct comparison to these models can be made with the bimodal results under scenario 2. All of the written values are accuracies and microaverage F1 scores. We write the best result for each dataset in bold.

The improvement when introducing a knowledge graph on the MACCROBAT dataset is substantially larger than on the other 2 datasets. We found that text based predictions on the dataset are less accurate than on the other datasets, as the examples are more difficult to predict. We believe that this allows the predictions based on a knowledge graph to improve recognized relations more than on other datasets, where the initial predictions are more accurate.

### Scenario 2: automatically inferred knowledge graph

Results in Scenario 1 demonstrate a significant performance improvement when combining text with a knowledge graph, compared to using the text model alone. However, this scenario assumes prior knowledge of document relations. In many real-world applications, we begin with unseen documents and aim to extract all temporal relations between events without additional information. To address this, we employ a text model to initially extract temporal relations from a document, using them to construct a knowledge graph. We also predict additional relations, such as transitive relations, to aid inference. We use the relations predicted between events from the same medical report. When predicting the initial relations, the model achieved an accuracy of 81% on the i2b2 dataset, 77% on the THYME dataset, and 76% on the MACCROBAT dataset on the expanded set of relations.

Subsequently, we utilize the constructed graph to enhance the final extraction of temporal relations. In this scenario, the knowledge graph may contain some incorrectly labeled relations due to the text model’s inherent inaccuracies. Our experiment results, detailed in Table 2 under Scenario 2, indicate that even when using the automatically constructed knowledge graph, performance improves compared to relying solely on the text model across all datasets, except the original THYME dataset. While the improvement is less than when using an event graph with accurate relations, this procedure can be applied to any document, even without prior extracted information.

### Scenario 3: ablation study

In section “Scenario 2: automatically inferred knowledge graph,” we observed that incorrect relations in the event graph notably affect the model’s performance. To investigate whether the primary source of performance degradation arises from the scarcity of correct relations or the presence of incorrect ones, we designed a new testing scenario. In this scenario, we initially constructed an event graph for each document using the text model, following the procedure outlined in section “Scenario 2: automatically inferred knowledge graph.” Subsequently, we used the target labels of the relations to identify and remove all incorrectly predicted relations from the graphs. The resulting graphs were then employed for temporal relation extraction, allowing us to assess the impact of incorrect relations on the model’s performance. It’s important to note that this scenario offers insights into the model’s performance and does not simulate a practical application, as it requires prior knowledge of the correct relations for the removal process.

The experiment results, as presented in Table 2 under Scenario 3, indicate that on the i2b2 and MACCROBAT datasets, using a combination of both modalities yielded an approximate 3% improvement over relying solely on the text model. This suggests that a substantial portion of the performance difference is attributed to the inclusion of incorrectly labeled relations in the graph. However, on the THYME dataset, the removal of incorrect relations did not lead to an improvement in results.

### Using softmax confidence to filter the event graph

**Figure 4.**
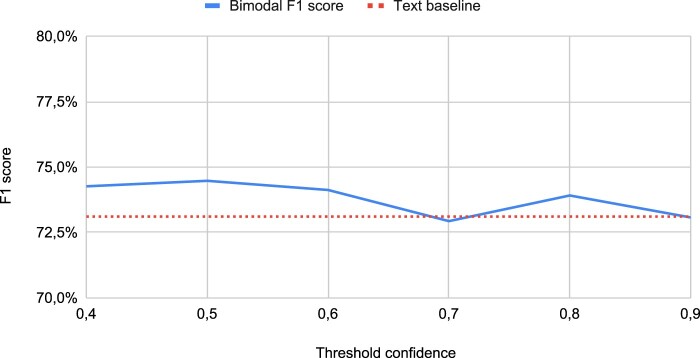
The F1 score as we change the threshold to filter predicted relations based on model confidence

We explored the potential for improving results by filtering relations with low softmax confidence. Our investigation focused on the i2b2 dataset. In this experiment, we applied various threshold values to filter the event graph, retaining relations with confidence scores exceeding the threshold. Multiple threshold values were tested, as displayed in Figure 4. The best performance was observed when using a threshold of 0.5. However, the improvement over not employing the threshold was marginal, at only 0.3%. Overall, filtering the event graph based on the confidence threshold did not yield a significant enhancement in predictions.

### Comparing the results to state-of-the-art models

In Table 2, we also included some models developed by the other authors to compare our model against. For comparison, we used results achieved by Ul Haq et al^29^ using BERT and BioBERT on the i2b2 dataset as well as the results achieved by Zhao et al,^30^ Wang et al,^31^ Tourille et al,^15^ and Leeuwenberg et al^32^ on the THYME dataset. We also compare the results to our results achieved by the Alpaca model and the architecture described by Lin et al,^20^ as their tests were done on a modified version of the THYME corpus.

We found that the proposed model outperforms state-of-the-art models on the i2b2 dataset under all scenarios while performing comparably to the model by Lin et al^20^ and outperforming the model by Zhao et al^30^ on the THYME dataset.

### Case study and error analysis

We manually selected representative examples from the i2b2 dataset to illustrate common scenarios observed during our model testing. To protect patient privacy, the sentences presented in this section have been synthetically generated to resemble examples from the dataset. While it can be challenging to discern the exact reasons behind the model’s predictions, we examine the predictions made by each component of the model separately and compare them to the final prediction. This approach provides insights into the factors influencing the ultimate prediction.

In Table 3, we present a sentence from the i2b2 dataset that contains 2 annotated relations involving 3 events. In this instance, the model made incorrect predictions for both temporal relations. The correct relation between “visited” and “painless jaundice” is ”overlap,” and the correct relation between “visited” and “weight loss” is “before.” For the first relation, both the text and graph components of the model agreed on predicting “after.” However, we note that based on the annotation guidelines,^33^ the sentence could also be interpreted as suggesting that painless jaundice occurs before the patient visits the Emergency Department. With this perspective, the model’s prediction could be considered correct, even if it diverges from the dataset annotation. In the second relation, the text component of the model disagrees with the graph component, leading to incorrect predictions from both parts and, consequently, an overall incorrect final prediction.

**Table 3.** An example of a sentence from the i2b2 dataset with annotated temporal relations.

The patient *visited*^1^ the ER with a complaint of *painless jaundice*,^2^ and *weight loss.*^3^		
Events	1→2	1→3
Correct label	O	B
Text	A	O
Graph	A	A
Bimodal	A	A

In this case, the model made a wrong prediction of both relations. Each event in the text is marked with a number. The relations are represented as O: overlap, A: after, and B: before.

In Table 4, features another example of a sentence containing 4 annotated relations. Notably, the text component of the model made accurate predictions for 3 of these relations, whereas the graph component correctly predicted only 1 relation. However, the model successfully integrated both sets of predictions in all instances, resulting in all 4 final predictions being correct.

**Table 4.** An example of a sentence with 4 relations, where the model correctly combined graph and text parts.

The patient was *admitted*^1^ to the *ICU*^2^ for *anemia*,^3^ and *seizures*^4^ immediately following the *surgery*5 but was then transferred back to the floor in stable condition				
Relation	2→3	2→4	2→5	1→5
True label	A	O	O	A
Text model	O	O	O	A
Graph model	A	A	A	O
Bimodal	A	O	O	A

Each event in the text is marked with a number. The relations are represented as O: overlap, A: after, and B: before.

## Discussion

In this study, we (1) introduced a novel approach that integrates text with a knowledge graph for temporal relation extraction. We (2) designed 3 testing scenarios and (3) evaluated our approach on multiple datasets and scenarios.

By leveraging additional temporal relations from the document as context for the model, we observed performance improvements across all datasets. Notably, the gains were most pronounced in the i2b2 2012 and MACCROBAT datasets. However, the improvement was less apparent when applied to the original THYME corpus. We believe that this is because the relations in the THYME corpus are more precise, so a small uncertainty about the times of the events can mean a completely different predicted relation. For example, if we know that event A *overlaps* with event B and event B *initiates* event C, the relation between events A and C could be *initiates*, *overlaps*, or *before*. This is also supported by the observation that the performance on the converted THYME dataset, where we combined types of relations into the 3 basic types (before, after, and overlap) is more in line with the other datasets.

A practical challenge arises when applying this approach to documents where no prior relations are known. To address this, we proposed initially building a knowledge graph by automatically recognizing relations based on the text. Using this extracted information as additional knowledge for the final model, we observed smaller performance gains, but still obtained 124 additional correct predictions (about 1% improvement) on the i2b2 dataset compared to using the text model alone. We believe this improvement stems from providing the model with a broader overview of all relations, rather than relying solely on the text surrounding a specific event.

Additional tests showed that filtering the automatically extracted graph to remove incorrect relations improves model performance. However, this filtering, dependent on knowledge of correct relations, is impractical for real-world use. Despite this, it highlights the potential benefit of eliminating potentially incorrect relations, even if it means excluding some correct ones. In section “Using softmax confidence to filter the event graph,” we explored using a confidence threshold and found that while low confidence filtering has marginal performance improvement, high confidence filtering reduces performance. To enhance this in the future, we plan to implement a system comparing predicted relations to those from other documents between similar events, assessing statistical likelihood. We believe combining statistical likelihood and confidence can offer a useful measure for prediction filtering.

One limitation of our work is that our linking library is constrained to using the event phrase without considering its context. This limitation can result in incorrect linking when events lack context. For example in the sentence “The patient was consented for both procedures and brought to the Operating Room.” the phrase “both procedures” was marked as 1 of the events in the text; however, the linker cannot know which events the sentence is talking about and links the phrase to the concept C0025664 (Methods aspects), which is a generic concept for all procedures.

Throughout our experiments, our bimodal architecture, which integrates textual information with a knowledge graph, demonstrated its capability to improve temporal relation extraction. Furthermore, we compared our results to models from other researchers and found that our model achieves state-of-the-art performance for the temporal relation extraction task.

## Conclusion

We introduce a novel multimodal architecture for temporal relation extraction, allowing the model to combine text and knowledge graph data. By incorporating additional temporal relations from the document, our model improves new temporal relation predictions, outperforming text-only approaches.

Our architecture is poised to advance temporal relation extraction in clinical text analysis, enabling the development of sophisticated automated tools for medical document analysis, trend identification, and pattern recognition across diverse patient records.

Future research will focus on leveraging comprehensive global knowledge graphs encompassing general medical concepts and patient-specific event instances. These graphs will enable effective information aggregation across patients while preserving local event structures, building upon the success of our current work.

## Data Availability

No new data were generated or analyzed in support of this research.

## Research data policy

No new data were generated or analysed in support of this research.
